# The genetic heterogeneity and mutational burden of engineered melanomas in zebrafish models

**DOI:** 10.1186/gb-2013-14-10-r113

**Published:** 2013-10-23

**Authors:** Jennifer Yen, Richard M White, David C Wedge, Peter Van Loo, Jeroen de Ridder, Amy Capper, Jennifer Richardson, David Jones, Keiran Raine, Ian R Watson, Chang-Jiun Wu, Jiqiu Cheng, Iñigo Martincorena, Serena Nik-Zainal, Laura Mudie, Yves Moreau, John Marshall, Manasa Ramakrishna, Patrick Tarpey, Adam Shlien, Ian Whitmore, Steve Gamble, Calli Latimer, Erin Langdon, Charles Kaufman, Mike Dovey, Alison Taylor, Andy Menzies, Stuart McLaren, Sarah O’Meara, Adam Butler, Jon Teague, James Lister, Lynda Chin, Peter Campbell, David J Adams, Leonard I Zon, E Elizabeth Patton, Derek L Stemple, P Andy Futreal

**Affiliations:** 1 Wellcome Trust Sanger Institute, Cambridge CB10 1SA, UK; 2 Memorial Sloan Kettering Cancer Center and Weill-Cornell Medical College, New York, NY 10065, USA; 3 Department of Human Genetics, VIB and University of Leuven, B-3000, Leuven, Belgium; 4 Delft Bioinformatics Lab, Delft University of Technology, Delft 2628CD, the Netherlands; 5 MRC Institute of Genetics and Molecular Medicine, MRC Human Genetics Unit and Edinburgh Cancer Research Centre, Edinburgh EH4 2XU, UK; 6 Department of Genomic Medicine, The University of Texas MD Anderson Cancer Center, Houston, TX 77054, USA; 7 Department of Electrical Engineering, University of Leuven, B-3001, Leuven, Belgium; 8 Dana Farber Cancer Institute and Boston Children’s Hospital, Harvard Medical School, Boston, MA 02115, USA; 9 Department of Human and Molecular Genetics, Virginia Commonwealth University, Richmond, VA 23298-0033, USA; 10 Institute for Applied Cancer Science, The University of Texas MD Anderson Cancer Center, Houston, TX 77030, USA

## Abstract

**Background:**

Melanoma is the most deadly form of skin cancer. Expression of oncogenic *BRAF* or *NRAS*, which are frequently mutated in human melanomas, promote the formation of nevi but are not sufficient for tumorigenesis. Even with germline mutated *p53*, these engineered melanomas present with variable onset and pathology, implicating additional somatic mutations in a multi-hit tumorigenic process.

**Results:**

To decipher the genetics of these melanomas, we sequence the protein coding exons of 53 primary melanomas generated from several *BRAF*^*V600E*^ or *NRAS*^*Q61K*^ driven transgenic zebrafish lines. We find that engineered zebrafish melanomas show an overall low mutation burden, which has a strong, inverse association with the number of initiating germline drivers. Although tumors reveal distinct mutation spectrums, they show mostly C > T transitions without UV light exposure, and enrichment of mutations in melanogenesis, *p53* and MAPK signaling. Importantly, a recurrent amplification occurring with pre-configured drivers *BRAF*^*V600E*^ and *p53*^*-/-*^ suggests a novel path of *BRAF* cooperativity through the protein kinase A pathway.

**Conclusion:**

This is the first analysis of a melanoma mutational landscape in the absence of UV light, where tumors manifest with remarkably low mutation burden and high heterogeneity. Genotype specific amplification of protein kinase A in cooperation with *BRAF* and *p53* mutation suggests the involvement of melanogenesis in these tumors. This work is important for defining the spectrum of events in *BRAF* or *NRAS* driven melanoma in the absence of UV light, and for informed exploitation of models such as transgenic zebrafish to better understand mechanisms leading to human melanoma formation.

## Background

Melanoma is a form of skin cancer known for its therapeutic resistance, aggressiveness and late metastatic manifestation [1]. Activating mutations in *BRAF* (V600E) or *NRAS* (Q61K) are collectively found in approximately 60% of human melanomas and result in the constitutive signaling of the mitogen-activated protein kinase (MAPK) pathway [2,3]. Although studies have shown a clear dependence of tumor growth on MAPK signaling, most nevi with *BRAF*^*V600E*^ or *NRAS*^*Q61K*^ mutations remain benign for decades [4]. In zebrafish, expression of human *BRAF*^*V600E*^ (*BRAF*) or *NRAS*^*Q61k*^ (*NRAS*) in melanocytes results in the growth of pigmented, nevus-like lesions that also rarely progress to melanoma. Invasive melanomas develop in these transgenic zebrafish only in combination with engineered loss of *p53* function [5,6], and yet manifest with variable onset and penetrance, strongly suggesting that these drivers are not sufficient for malignant melanoma formation and the requirement for additional unknown, somatic events.

Recent analyses of the genomes and exomes of human melanoma have resulted in the identification of new mutations that are likely to contribute to the disease formation or survival [7-11]. One confounding aspect of discriminating drivers in melanoma is the elevated background mutation burden due to UV mutagenesis, although new algorithms have been developed to refine this analysis [10]. We sought to build upon these studies through a focused analysis of a set of engineered melanomas, to determine the spectrum of mutations in the absence of UV light and to interrogate the role of *BRAF*, *NRAS* and *p53* in melanoma in transgenic zebrafish. Specifically, we used targeted exon enrichment and Illumina sequencing to generate exome and copy-number alteration data for 53 samples consisting of 38 *BRAF*-driven and 15 *NRAS*-driven primary zebrafish melanomas and cell lines with additional perturbations. A detailed examination of the spectrum of somatic point mutations, insertions, deletions and amplifications is presented. Our analysis reveals striking genetic heterogeneity, genotype-specific mutation patterns and a potential novel path to *BRAF*-driven tumorigenesis, providing insights into the events important for cooperation with *BRAF* and *NRAS* in the context of low mutation burden.

## Results and discussion

### Study set and sequencing overview

**Figure 1:**
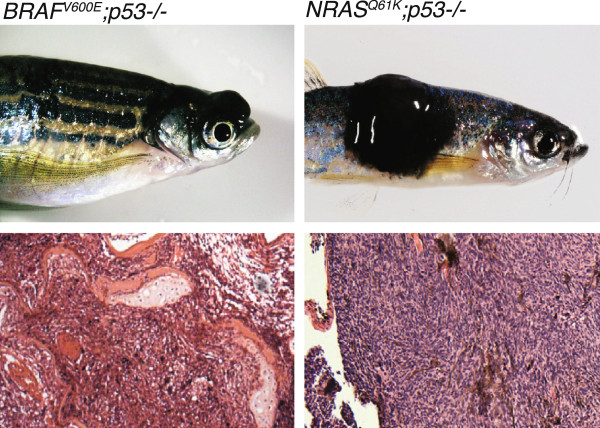
**Examples of zebrafish melanomas.***BRAF* (left panel) and *NRAS* (right panel) driven zebrafish melanomas in a *p53*^-/-^ background, with specimen example (top panel) and histology (bottom panel).

We collected matched zebrafish melanoma and normal tissue from 53 transgenic zebrafish harboring tissue-specific oncogenic alleles of human *BRAF* and *NRAS* under a melanocyte-specific (*mitf*) promoter [5,6] (Table 1, Figure 1; Additional file 1: Table S1). Specifically, 38 fish expressed oncogenic *BRAF*^*V600E*^ (*BRAF*) and 15 expressed oncogenic *NRAS*^*Q61K*^ (*NRAS*). The majority of samples (33 *BRAF* and 14 *NRAS* individuals) carried at least one germline, mutant *p53* allele (*p53*^*M214K*^[12]). While *p53* itself has not traditionally been considered to be a major tumor suppressor in melanoma development, inactivation of *CDKN2A/p16* is associated with loss of *p53* activity [13]. Further, the high mutation load in *p53* and its pathway components in melanoma also underscores its importance [10]. Four *BRAF* fish harbored a germline temperature-sensitive hypomorphic allele of *mitf* (*mitf* ^*vc7*^) [14,15]. Of *BRAF* individuals with aberrant *p53*, 38 had additional mutant germline alleles in *mitf* ^*-/-*^ (known as *nacre*^-/-^) [16], *ptena*^*hu1864 +/-*^[17] or *mitf* ^*vc7*^[14,15]. Transgenic individuals with *BRAF;p53*^*-/-*^*;mitf* ^*-/-*^ were manipulated with a miniCoopR shuttle vector system [18], consisting of somatic mosaic rescue of *MITF* expression in melanocytes along with *SETDB1*[18] and transcription factors *KROX20*, *FOXD3* or *OCT6*, the biology and oncogenicity of which are being investigated independently.

**Table 1:** Study set overview

***Genotype***	**Samples**
*mitf:BRAF*^*V600E*^	1
*mitf:BRAF*^*V600E*^*;p53*^*+/-*^	2
*mitf:BRAF*^*V600E*^*;p53*^*+/-*^*; ptena*^*hu1874*^^*+/-*^	1
*mitf:BRAF*^*V600E*^*;mitf* ^*vc7+/+*^*;*	4
*mitf:BRAF*^*V600E*^*;mitf* ^*vc7+/+*^*;p53*^*+/-*^	4
*mitf:BRAF*^*V600E*^*; p53*^*-/-*^*;mitf* ^*-/-*^*;mitf:MITF*	6
*mitf:BRAF*^*V600E*^*;p53*^*-/-*^*;mitf* ^*-/-*^*;mitf:MITF;mitf:foxd3*	4
*mitf:BRAF*^*V600E*^*;p53*^*-/-*^*;mitf* ^*-/-*^*;mitf:MITF;mitf:krox20*	12
*mitf:BRAF*^*V600E*^*;p53*^*-/-*^*;mitf* ^*-/-*^mitf:MITF;mitf:krox20/foxd3/OCT6*	1
*mitf:BRAF*^*V600E*^*;p53*^*-/-*^*;mitf* ^*-/-*^*;mitf:MITF;mitf:SETDB1*	1
*mitf:BRAF*^*V600E*^*;p53*^*-/-*^*;mitf* ^*-/-*^*;mitf:MITF;mitf:EGFP*	1
*mitf:NRAS1*^*Q61K*^	2
*mitf:NRAS1*^*Q61K*^*;p53*^*-/-*^	5
*mitf:NRAS1*^*Q61K*^*;p53*^*+/-*^	4
*mitf:NRAS1*^*Q61K*^*;p53*^*+/-*^*;rps29*^*+/-*^	5
*Total*	53

The asterisk indicates that the genes *krox20*, *foxd3* and *OCT6* were each expressed on separate plasmids for this tumor.

To analyze coding regions of the zebrafish genome, we performed targeted exome capture on tumor and normal DNA followed by 75 base paired-end Illumina (HiSeq) sequencing (European Nucleotide Archive accessions ERP003701, ERP003702). The bait set covered all protein coding genes, 3’ UTRs and 5’ UTRs of the Zv8 and later Zv9 genome for a combined coverage of 60 Mb. A total of 2,309 Gb of sequencing was generated, averaging approximately 21.8 Gb per sample (Additional file 1: Table S2).

Because of the complexity and diversity of the zebrafish genome [19], we addressed the sensitivity and precision of applying the CaVEMan substitution calling algorithm [20] to zebrafish through two analyses: variant calling simulations and comparison to additional callers. In the first instance, we measured the performance of CaVEMan in simulated zebrafish tumor and normal genomes, which showed that the algorithm detected somatic substitutions with both high sensitivity and precision within these conditions (Additional file 2: Figure S1, Supplementary text in Additional file 3). We next employed CaVEMan for substitution calling on the zebrafish melanoma study set. Through manual inspection of each variant, we determined that a large proportion of these substitutions were false positives (57%; Additional file 2: Figure S2A), many due to germline variants that had been missed by the algorithm or calls made on suboptimal alignments (Additional file 2: Figure S2B). The low precision led us to manually examine all variants to ensure an accurate collection was used for downstream analysis.

In the second part of the analyses, we ascertained the sensitivity of our algorithm on the zebrafish melanoma dataset by comparing the CaVEMan calls for one sample (ZD8a) to those from SomaticSniper [21] and String Graph Assembler (SGA) [22]. Our results showed that SomaticSniper, and not SGA, provided a 10% increase of somatic variants to the CaVEMan algorithm (Additional file 2: Figure S2C-E). In spite of this marginal increase, we added a subset of non-overlapping Sniper variants to the CaVEMan calls, which we experimentally validated through targeted enrichment and Illumina sequencing (Additional file 2: Figure S3). All calls from this analysis were then subject to a second, manual review.

### Overview of substitutions and indels in engineered zebrafish melanomas

**Figure 2:**
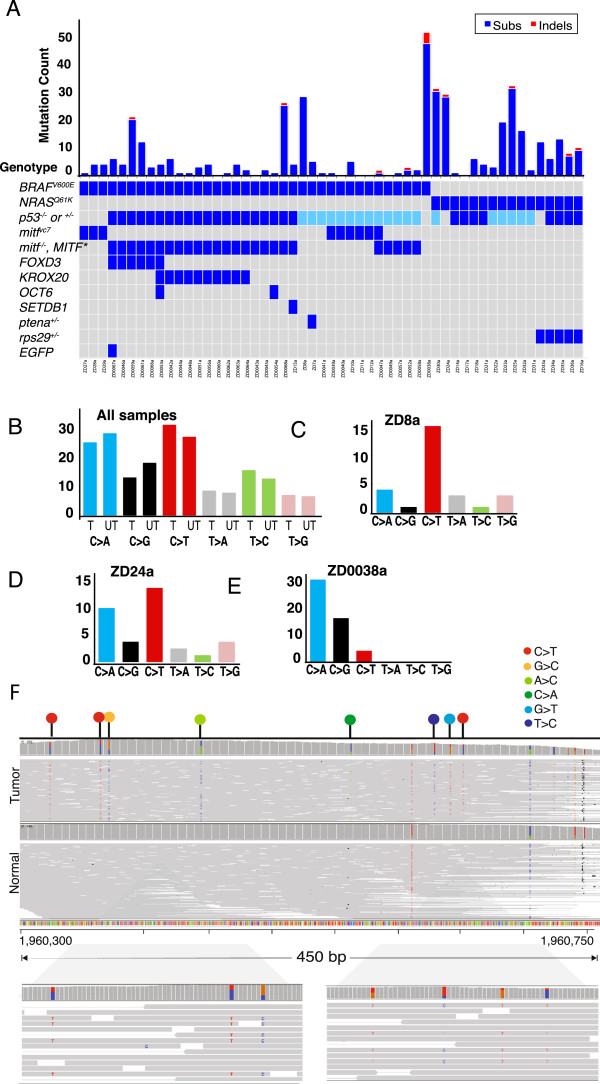
**Overview of substitutions. (A)** The number of substitutions (dark blue columns) and indels (red columns) per sample, corresponding to their initiating germline mutations (bottom shaded). For *p53*, light blue indicates *p53*^*+/-*^ and dark blue *p53*^*-/-*^. Asterisk specifies *mitf:MITF* expression in a *mitf* ^*-/-*^ background. **(B-E)** Mutation spectrum of all and selected samples. For all samples **(B)** mutations are indicated on the transcribed (T) and untranscribed (UT) strand. **(F)** Evidence of kataegis within 4,500 bp region in ZD8a, a *BRAF;p53* mutant sample. Somatic mutations are highlighted with colored circles corresponding to the type of substitution.

We confirmed a total of 403 point mutations and 13 insertions and deletions (indels), the latter of which were identified using Pindel [23] and processed using a similar method to the substitutions (Figure 2A). Of the substitutions, 79 were synonymous, 168 resulted in amino acid changes, 16 were nonsense and 25 occurred at splice sites (Additional file 1: Table S3). Eighty-five substitutions were found in the 3' UTR and 26 in the 5' UTR, and one start codon was gained. The ratio of 2.3:1 non-synonymous to synonymous events was similar to the averages previously reported in human melanoma [9,10]. The median number of coding mutations per sample was four, significantly fewer than the median of 171 in sun-exposed human melanomas and closer to the median of nine in mucosal and uveal melanomas, also originating from sun-shielded sites [9]. Over half of the total number of mutations in the study set was present in only eight samples (15%), six of which had two or fewer engineered ‘initiating drivers’. The highest number of substitutions were found in samples with one or two initiating drivers: ZD0038a (*BRAF*), ZD24a (*NRAS*), ZD23a (*NRAS;p53*^*+/-*^) and ZD30a (*NRAS;p53*^*-/-*^).

Consistent with the low substitution burden, there were few recurrent mutations. Two substitutions were found in *ttna* and *ttnb*, the two largest protein-coding genes in the zebrafish genome. No recurrent substitutions were found in known melanoma genes or genes in the Cancer Gene Census [24]. Over 60% of genes mutated in this study were found to be mutated at least once in human melanoma [9-11,25-27], which was unsurprising given the extensive mutation load in the human disease. Substitutions with predicted coding changes in known census cancer genes included a nonsense mutation in *ikzf* and missense mutations in *nup214* and *pik3cd*, while a homozygous missense substitution in the anaphase promoting complex gene, *anapc1*, was identified in a *BRAF*, *p53*^*+/-*^ tumor (ZD8a).

### UV-independent mutation spectra and mutational processes

Intriguingly, recent studies have shown that over half of the driver mutations in human melanomas do not bear the UV radiation-associated signature [10]. To explore the nature of the non-UV events, we examined the mutation spectrum in the engineered zebrafish melanomas developed under conditions without detectable UV light, as determined using a standard laboratory photometer (International Light 1400). As with most human cancers, C > T substitutions (24.4%) constituted the prominent mutation class across all samples, including ZD8a and ZD24a (Figure 2C,D), which had substantial mutation burdens. Remarkably, ZD0038a, which had the highest substitution load (n = 47), consisted of mutations occurring exclusively at cytosine or guanine residues (Figure 2E), a mutation signature that has not yet been described in human cancers. In this sample, all coding substitutions apart from one resulted in a predicted missense (n = 21) or a nonsense change (n = 3). To determine if this was the result of positive selection, we calculated the dN/dS ratio using a mutation-selection model. We found that the rates of missense and nonsense mutations for this sample were approximately 5.5 and 9.8 times higher than expected by neutral evolution, respectively, a result unlikely in the absence of positive selection (*P*_*dM/dS*_ = 0.030 and *P*_*dNS/dS*_ = 0.031).

Similar to findings in non-sun-exposed human melanomas [9], no significant bias of mutations was found in any class on any particular strand (Figure 2B). By comparison, a mutation strand-bias caused by transcription-coupled repair has been demonstrated in UV light-induced melanomas, lung and breast cancers, all of which display the characteristic signatures of their respective UV, tobacco and DNA repair mutagens [9,28,29]. The absence of this signature in our samples suggests that these repair processes are not overt unless triggered by a selective, mutagenic pressure.

ZD8a, a *BRAF* and *p53* mutant, presented two microclusters of mutations. Twelve substitutions (40% of the total load) spanned exons within a 4,500 bp interval of the *hoxd9a* and *hoxd10a* genes (Figure 2F), while a second cluster of five mutations was found within a 5 kb interval (Additional file 2: Figure S4). These microclusters were reminiscent of ‘kataegis’, hypermutated regions resulting from a single event [30]. A close examination of the reads revealed that the substitutions occurred in *cis*, had similar variant allele fractions and were mostly C > T transitions (n = 12/26; Figure 2F). In human, patterns of kataegis have been proposed to be related to mutational processes of the AID/APOBEC family of enzymes, which modulate antibody diversification by deaminating cytidines to deoxyuridine within immunoglobulin genes [30,31]. Although APOBEC emerged only in primates, they are believed to have derived from the functionally conserved AID enzymes [32], which may provide the mechanistic origin of these clusters in zebrafish.

### Insertions and deletions

Indels were sparse, with a total of 13 confirmed indels across the 53 samples (Additional file 1: Table S4). This is lower than the sample average of two to four indels in human melanoma [9]. Eight indels were single base pair indels, and all 13 (<5 bp) were flanked by tandem repeat sequences on either side, evidence of a lapse in post-replicative mismatch repair found commonly in breast cancer genomes [30]. Ten indels were out of frame and likely to cause loss of gene function. Four indels (36%) were found in a sample mutant only in *BRAF* (ZD0038a). Interestingly, a single nucleotide deletion resulting in a frameshift mutation was found in *pik3ip1* (V170fs*), which in human directly binds to the p110 catalytic subunit of PIK3 and negatively modulates its activity [33]. Its occurrence in a *BRAF*, *mitf* ^*-/-*^, *p53*^*-/-*^ mutant sample is consistent with a role for phosphatidylinositide 3-kinase (PI3K) cooperation with MAPK deregulation in human melanoma [34].

### Overview of copynumber changes

In total, 991 amplification segments (copy number ≥5 for samples with ploidy <2.7, and copy number ≥8 for samples with ploidy ≥2.7) and 436 segments of homozygous deletions (copy number = 0) were identified by ASCAT [35]. There was marked variation in the number of copy number changes among samples in the study set, with a cumulative 5 Gb of losses or gains manifesting in over half of tumors analyzed. For samples represented by both array comparative genomic hybridization (aCGH) and ASCAT data, the frequency recurrence profiles of copy number changes from ASCAT generally agreed with those from aCGH performed on the same DNA stock (Additional file 2: Figure S5).

**Figure 3:**
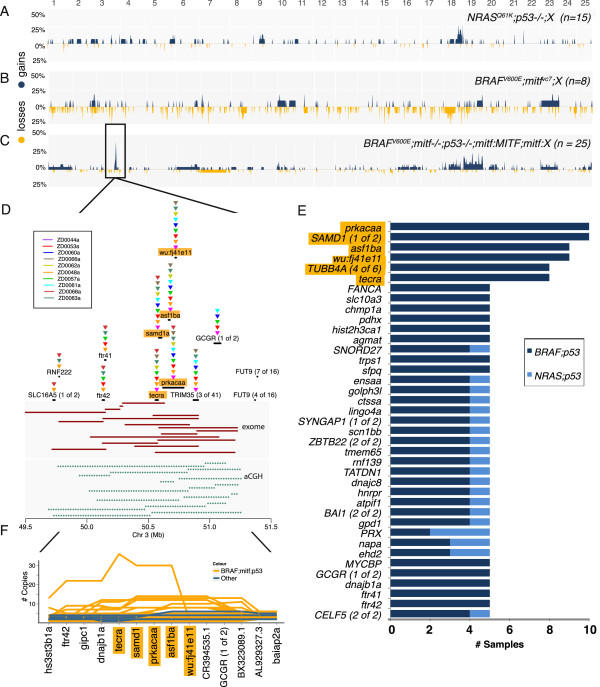
**Identification of a frequently amplified locus on chromosome 3.** Frequency profiles of tumors mutant in **(A)***NRAS;p53*^*-/-*^*;X*, **(B)***BRAF;mitf* ^*vc7*^*;X* tumors and **(C)***BRAF;p53;mitf* ^*-/-*^*;mitf:MITF;mitf:X*, where *X* can include additional drivers as mentioned in the text. **(D)** Amplification segments supporting a peak on chromosome 3 in tumors of *BRAF;p53;mitf* ^*-/-*^*;mitf:MITF;mitf:X* background derived from exome sequencing (maroon segments) and aCGH (green dotted segments). Samples mutated are represented by inverted, color-coded triangles above the corresponding gene indicated by the thick black bar. **(E)** Frequently amplified genes in the entire dataset. **(F)** Number of copies (y-axis) of the genes (x-axis) in the region of amplified locus. Each line represents a tumor that is color-coded according to either *BRAF;p53;mitf* ^*-/-*^*;mitf:MITF;mitf:X* (yellow) or other (blue) background status. The most frequently amplified genes are highlighted in yellow in **(D-F)**.

While the majority of samples (85%) harbored at least one amplification, only 30% of the samples showed any homozygous deletions (Additional file 1: Table S5). It is therefore worth noting that *BRAF*-driven tumors mutant in *mitf* ^*vc7*^ had significantly more homozygous deletions than expected by chance (*P* = 0.01 by Chi-Square test; Figure 3B). *NRAS* subtypes, by contrast, did not reveal apparent commonalities (Figure 3A). Clustering of ASCAT and aCGH segments from all samples also did not reveal any regions of subgroup affiliation apart from the strong amplified signal on chromosomes 18 and 19 (Additional file 2: Figure S6), the latter of which is believed to be associated with the *BRAF*^*V600E*^ transgene integration as a concatemer on chromosome 19.

### Identification of a recurrently amplified region in a subset of zebrafish melanomas

A particularly striking finding was the recurrence of a 175 kb amplicon on chromosome 3 (50.0 to 51.2 Mb) in 10 tumors belonging to the *BRAF*, *p53*^*-/-*^, *mitf* ^*-/-*^ background with *MITF* rescue (Figure 3C). Although this subgroup is the largest of our dataset (47%, 25/53), the clustering of the recurrent amplicon in this subgroup was unlikely to have occurred by chance (*P* = 0.000256 by Chi-Square test). Amplified segments were supported by both ASCAT and aCGH (Figure 3D). The most frequently amplified genes were *prkacaa* and *samd1* (1 of 2), presenting in 10/53 samples, followed by *as1ba* (n = 9), *wu:fj41e11* (n = 9) and *tecra* (n = 8) (Figure 3E). While amplifications were found across all 10 samples for *samd1* and *prkacaa*, they presented in five or fewer samples for flanking genes *RNF222* and *gcgr* (Figure 3D).

A simulation was performed to determine the likelihood of the events occurring in these genes, at this frequency, by chance. For each sample, the number and lengths of the amplified segments were randomly introduced across the target exome regions one million times, producing a *P*-value that was adjusted by Bonferroni correction (n = 6,677). We did not factor causes of amplification other than those by chance, such as nearby fragile sites, for which little information is available for zebrafish.

Based on our simulations, all genes recurrently amplified in six or more samples were likely to be significant, including *prkacaa*, *samd1*, *asf1ba*, *wu:fj41e11* and *tecra* (n = 13; Additional file 1: Table S6). These genes did not show evidence of amplification or overexpression in human cancer datasets (CCLE, Oncomine, COSMIC) or large, comprehensive melanoma studies [9,10]. Genes recurrently amplified in fewer samples also showed significant enrichment (*P* ≤ 0.05; Additional file 1: Table S6). Among these, interestingly, was *tert* (*P* = 0.0, n = 4 samples), which encodes the reverse transcriptase subunit of telomerase responsible for maintaining the ends of chromosomes. *Tert* was the only known cancer gene recurrently mutated in our study set. In human melanoma, *TERT* is amplified [10,36] and harbors promoter mutations in as many as 90% of melanoma cases [7,8].

### Identification of few recurrent homozygous deletions

**Figure 4:**
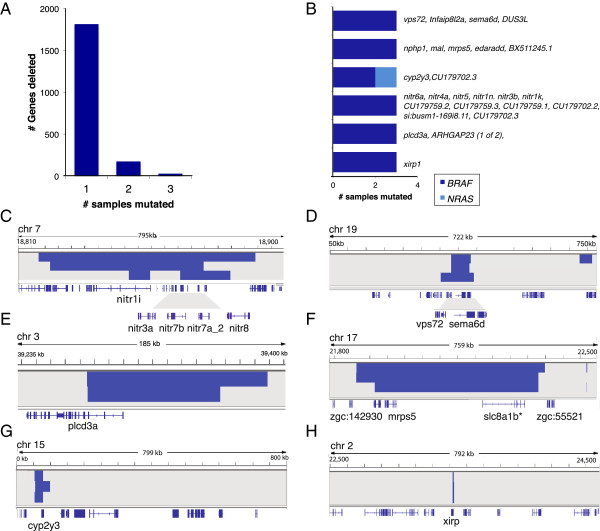
**Overview of homozygous deletions. (A)** Frequency of homozygously deleted genes across samples. **(B)** Recurrently deleted loci occurring in at least three samples that are driven by *BRAF* (dark blue) or *NRAS* (light blue), and the corresponding genes in these regions (right-hand side). **(C-H)** Examples of deleted segments (dark blue bars) and the genes in these regions (labeled at the bottom), represented by their exon structures (dark blue lines).

A total of 366 deletion events were identified, affecting the same genes in at most three samples in the study set (Figure 4A). By performing the above simulations, we determined the majority (28/30) of genes deleted in three samples were unexpected by chance (Additional file 1: Table S7). The genes *nitr1i*, *nitr3a*, *nitr7b* and *nitr7a* were in a locus deleted in three samples belonging to both *BRAF* and *NRAS* mutant lines (Figure 4B). The *nitr* genes are members of a highly diversified, multigene family of novel immune type receptor found in teleosts. *Nitr* genes do not rearrange like immune receptors but show structural similarities to both the mammalian T-cell or Ig-like receptors [37,38]. Loss of these genes could be relevant to one facet of progression, which is to avoid immune surveillance, consistent with a critical role of immune regulation in human melanoma [39]. Other recurrently deleted genes include *sema6d*, *plcd3a*, *mrps5*, *cyp2y3* and *xirp* (Figure 4C-H), none of which had been previously implicated in human cancer. Further investigation would provide insights into the contribution of these genes to tumorigenesis in zebrafish.

### Relationships between age, drivers, and mutation burden

We exploited the model system to explore the footprints of mutagenesis assuming a uniform basal mutational clock. Where data for the age of fish (at tumor collection) was available, we found a significant correlation between age and the number of substitutions using the Pearson’s correlation test and a generalized linear model (GLM) (R = 0.37, *P* = 0.02, GLM *P =* 0.0035). Positive correlations have similarly been found in human melanoma [9]. Age of onset and number of drivers were themselves strongly negatively correlated. If each germline driver was counted as one event in a requisite multistep process, we could attempt to delineate a relationship between these initiating events and extent of the mutations. For transgenic zebrafish of the genotype *BRAF*^*V600E*^*;p53*^*-/-*^*;mitf* ^*-/-*^*;mitf:MITF*, a value of four drivers was assigned, due to the yet unknown contributions of the additional genes (*KROX20*, *FOXD3*, *OCT6*) to melanoma. Interestingly, our data showed a significant, inverse association between the number of drivers and the substitution events (R = -0.45, *P* = 0.00075, GLM *P* = 0.00031), indicating that a greater number of drivers require fewer additional events to generate the melanoma lesions. To determine if this correlation extended to copy number events, we considered each amplified or deleted segment as an event in the tumor. Our data showed that if we considered drivers and age together, this was also a significant predictor of the total number of copy number events (GLM *P* = 0.00011; Additional file 1: Table S8).

### Functional categorization of frequently mutated genes

Similar to human cancers, the engineered melanomas overall displayed high heterogeneity, where the majority of genes mutated in only one sample (68%; Additional file 2: Figure S7A). Taking into account all the different modalities of mutation and their frequencies of occurrence, a *P*-value was calculated (using a binomial test) for each mutated gene (Additional file 1: Table S9). Due to the high frequency of recurrence, *prkacaa* and *samd1* presented with the highest significance (*P* = 2.31 × 10^-8^). Following this, we explored the potential functional themes underlying these aberrations through a KEGG (Kyoto Encyclopedia of Genes and Genomes) pathway analysis, which revealed that the enrichment for most pathways declines when the minimum threshold for number of mutated samples is raised (Additional file 2: Figure S7B). From this we infer that although many genes are not frequently mutated and significant by themselves (that is, mutated only once), the pathways in which they reside are significantly mutated. Among the enriched pathways in this study (Additional file 1: Table S10), two include biological processes that have been directly linked to the hallmarks of cancer (apoptosis and vascular endothelial growth factor signaling, for angiogenesis), while deregulation of two other pathways, p53 signaling and melanogenesis (Additional file 2: Figure S7C), have previously been implicated in melanoma [40]. Also showing significant enrichment was the MAPK signaling and cell cycle pathway, thus further supporting functions important in human melanoma development.

## Conclusions

We have provided a comprehensive overview of the genetic events in engineered zebrafish models harboring known driver alleles. Several new insights into the mutagenic processes in non-UV-mediated, engineered melanomas, and the biology of *BRAF* and *NRAS-*driven malignancies, can be drawn from these 53 exomes.

Our results show that in the absence of direct UV light, engineered melanomas develop similar mutational signatures to most human cancers, dominated by the evolutionarily conserved spontaneous deamination of cytosine to thymidine [9]. We also found rare cases exhibiting remarkably distinct mutation spectra, including indications of kataegis and a novel, unclassified mutational signature.

Importantly, our results demonstrate that tumors driven primarily by a greater number of known cancer genes typically manifest with fewer mutations, suggesting that such models can be used to bound and estimate the number of events in human cancers. Mouse models of acute myeloid leukemia and mammary tumors similarly displayed fewer mutations and structural rearrangements, respectively, than their human counterparts [41,42]. It could be speculated that predisposed human individuals would support the same conclusion. With nearly half of the samples presenting no substitutions or indels, however, these two classes of mutations are unlikely to be the only route to the additional mutations needed for full melanoma development, with potentially other factors such as chromatin modifications at play.

The highly recurrent amplicon in transgenic lines with *BRAF;p53;mitf* ^*-/-*^*;mitf:MITF* encompassing the genes *prkacaa*, *samd1*, *tecra*, *wu:fj41e11* and *asf1b*, indicates a strong selection for genes in this interval in mutant *BRAF*, *p53* and *mitf* lines with *MITF* rescue. Although the amplicon was exclusive to the *BRAF;p53;mitf* ^*-/-*^;*mitf:MITF* transgenic models, it is unclear whether it would also present in the *BRAF* or *BRAF;p53* mutant models given a larger sample cohort. Since *MITF* serves as a functional rescue in this transgenic line, the genetics of this subset may be comparable to human tumors that show dependency on *MITF* for growth, either through *MITF* amplification or overexpression. That none of the genes in this amplicon have been reported so far as mutated in human melanoma could therefore be due to its specific occurrence with *BRAF*, *p53* and amplified *MITF*, found in less than 5% of *BRAF* mutant metastatic melanomas and a rare combination (Additional file 2: Figure S8).

In this amplicon, amplification of *prkacaa*, which encodes one of two principal catalytic (C) subunits of protein kinase A (*pka*), is intriguing for several reasons. Human *PRKACA* is the principle catalytic subunit of protein kinase A (PKA) [43]. Although not previously associated with melanoma, the cAMP-PKA pathway is a major signal transduction pathway for melanin production, melanocyte proliferation and differentiation (reviewed in [44]) and has been implicated in pituitary tumorigenesis [45,46]. Mutations in *PRKAR1A*, a PKA-regulatory subunit, cause an inherited syndrome called the Carney complex, characterized by pigmented skin lesions, schwannomas, recurrent mucocutaneous myxomas and endocrine neoplasms [47,48]. Indeed, cAMP-dependent PKA activation has been shown to result in the upregulation of the *mitf* promoter, tyrosinase expression and melanin synthesis, affecting skin pigmentation and melanogenesis [49]. Of interest, recent data have interestingly demonstrated a link between pigment production and UV-independent melanomagenesis, where harmful accumulation of pheomelanin intermediates or by-products during pigment synthesis can promote tumor formation [40]. Thus, a potential consequence of *PRKACA* amplification may be disruption of PKA signaling and pigment production, pointing to its possible contribution to aberrant pigment production in UV-independent carcinogenesis.

An important observation of this study is that, apart from the amplicon, the *BRAF*- and *NRAS*-driven melanomas display striking genetic heterogeneity similar to human cancers and mouse cancer models [41,42]. One interpretation of this finding is that tumorigenic processes are achieved through the contribution of many different mutated genes, in line with previous findings in mice cooperativity screens [50] and low frequency drivers unveiled from emerging studies of human melanoma [9-11]. The enrichment of mutations in pathways known to be important for melanoma development, such as *MAPK* and *p53* signaling, in the presence of germline mutations affecting *BRAF*, *NRAS* and *p53*, also suggest that further modulation of the signaling of these pathways is required for full manifestation of the tumors.

To the best of our knowledge, the spectrum of somatic coding mutations in an engineered model of melanoma has not yet been described. The integrated analysis we report here thus provides a glimpse into the genetic paths to *BRAF*- and *NRAS*-driven tumorigenesis, providing a framework for genomic characterization, and a standard for evaluating and prosecuting detailed biological questions in engineered animal models of cancer.

## Materials and methods

### Simulation of zebrafish cancer genomes

Individual zebrafish genomes were created with a SNP density of 0, 0.001, 0.01, 0.1, and 0.5 SNPs/base by randomly generating substitutions across the genome using an in-house simulation script. Using each individual genome, referred to as the ‘normal’, we created a second genome containing an additional 2,000 substitutions for the ‘tumor’. For each normal and tumor genome, we simulated 75 bp reads in FASTQ format using wgsim [51], specifying null for the base mutation rate, error rate and indel mutation rate. To simulate normal contamination, we combined normal and tumor FASTQ files for each individual according to the following proportions to obtain an average sequencing coverage of 80× (Table 2).

**Table 2:** Metrics for simulating normal contamination in tumor and normal genome FASTA files

**SNP density**	**Type**	**Millions of reads**			
		**Normal**	**Tumor content**		
			**30%**	**60%**	**100%**
0	Normal	8	5.6	3.2	0
	Tumor	0	2.4	4.8	8
0.001	Normal	8	5.6	3.2	0
	Tumor	0	2.4	4.8	8
0.01	Normal	8	5.6	3.2	0
	Tumor	0	2.4	4.8	8
0.1	Normal	8	5.6	3.2	0
	Tumor	0	2.4	4.8	8
0.5	Normal	8	5.6	3.2	0
	Tumor	0	2.4	4.8	8

The simulated tumor and normal pairs were subsequently processed through the Cancer Genome Project Sequencing Pipeline.

### Sample collection

Zebrafish tumor and normal tissue samples were obtained from Amy Capper and Jennifer Richardson (Elizabeth Patton’s lab, University of Edinburgh, Edinburgh, UK), and from Richard White and Charles Kaufman (Len Zon’s lab, Boston Children’s Hospital, Boston). All samples were obtained in accordance with the UK Home Office regulations, UK Animals (Scientific Procedures) Act 1986, and reviewed by the Wellcome Trust Sanger Institute Ethical Review Committee. Samples from Elizabeth Patton’s lab were subject to histopathological review by a clinical pathologist (Marie Mathers, Edinburgh Western General Hospital). We were unable to perform histopathology on samples from Len Zon’s lab. Normal tissue included sections from the fin, head, or gut. Zebrafish melanoma and normal DNA were extracted from fresh frozen tissues using the Qiagen Blood and Tissue DNAeasy Kit (catalogue number 69504 (Hilden, Germany)). Melanomas were derived from transgenic zebrafish expressing either the *BRAF*^*V600E*^ or *NRAS*^*Q61K*^ human oncogene as previously described [5,6,18].

### Exome bait set

Exon sequences for bait set design were initially downloaded from BioMart [52] to encompass all protein coding genes, and 3’ UTR and 5’ UTR regions from Ensembl 58 of the Zv8 genome. The bait set was subsequently adjusted to encompass additional genes from Ensembl 61 and new releases of the Zv9 genome (Zebrafish Agilent All Exon SureSelect). A total of 2,309 Gb of sequencing was generated, averaging approximately 21.8 Gb per sample, of which 79.6% of reads mapped and 55% of which mapped to target coding regions (that is, ‘on target’; Additional file 1: Table S2). By comparison to the human exome [20], the performance of the zebrafish exome was slightly lower (in human, 89% of reads map, averaging 62% on target coverage), requiring a greater total sequencing depth to acquire the desired baseline coverage of 20 ×.

### DNA and library preparation, capture and sequencing

DNA libraries were prepared using the Illumina Paired End Sample Prep Kit according to the manufacturer’s protocol. For targeted enrichment, in the first iteration, we designed a custom bait set to target the zebrafish exome for solution capture to include all the exons of all protein coding genes in the Zv8 Ensembl 58 gene build. Subsequently, an additional 2,059 genes were added to include improved annotations in the Zv9 assembly and Ensembl 59 gene build. Targeted enrichment was performed as described [53] following the manufacturer’s instructions.

Sequencing with 75 base paired-end reads of targeted-enrichment libraries was performed on the Illumina GAIIx and the HiSeq 2000 sequencers. Reads were mapped to the zebrafish reference (Zv9 Ensembl 61) using the Burrows-Wheeler algorithm (BWA version 0.5.9) [54] under default parameters and excluding library PCR duplicates.

### Identification of substitution variants

#### CaVEMan

CaVEMan (cancer variants through expectation maximization), an in-house algorithm, was employed to call single nucleotide substitutions in our dataset. Post-processing filters developed for human variant calling and additional filters were applied to the set of initial CaVEMan mutation calls to improve the specificity of the output.

#### SomaticSniper

Tumor and normal BAM files were processed by SomaticSniper [21] with a specification for read and base quality of at least 40. Raw variants were post-processed using scripts obtained through Github [55], modified to include a variant allele frequency of no more than 3% in the normal sample and less than 10% of the tumor, and without germline SNPs or indels within 5 bp of any of the normal zebrafish exomes. Variants were annotated using the Ensembl variant effect predictor (Ensembl 64 gene build) specifying only coding variants as output.

#### SGA

SGA analysis was run by Jared Simpson using a modified algorithm [22].

### Identification of insertions and deletions

Insertions and deletions were called using a modified version of Pindel [23] as previously described [28]. To improve the identification of high confidence variants, we specified a requirement for a minimum depth of 15 reads in both tumor and normal samples. For small indels, at least four reads supporting the variant seen by Pindel and at least one by BWA were required. Larger indels were defined in non-repeat regions where the mutation was seen once on either strand by Pindel. All indels were manually reviewed for confirmation.

### Variant validation

#### Capillary and 454 resequencing

Validation of substitutions and indel variants was initially attempted through capillary or 454 Roche resequencing of amplified PCR products spanning the mutation in the tumor and the normal DNA, which had been subject to whole-genome amplification from the original stock using GenomiPhi (illustra GenomiPhi HY DNA Amplification Kit, catalog number 25-6600-20 (Little Chalfont, Buckinghamshire, United Kingdom)), according to the manufacturer’s instructions. Nested PCR improved PCR yield over a single round of amplification, but both capillary and 454 Roche approaches proved problematic in PCR-amplified zebrafish DNA.

#### Targeted capture and Illumina sequencing

To circumvent problems with PCR-based validation, we designed a custom bait set targeting the mutant alleles for enrichment followed by Illumina sequencing. We streamlined the validation study set by qualitatively reviewing each variant and keeping only CaVEMAN calls that did not show germline mutations and were supported by high quality mapping reads and alignment. An additional 1,700 non-overlapping, Sniper variants (60% of the total non-overlapping Sniper calls) were selected at random to include in the validation set, comprising a bait set of 1.4 Mb with minimal tiling probes flanking 60 bp on either side of each variant. DNA libraries were made as described above and pooled into eight samples per group with barcode identifiers. Targeted capture was performed with each pool according to manufacturer’s instructions followed by 100 bp paired-end sequencing on the Illumina HiSeq 2000 and default BWA alignment. Mutant variants were confirmed on Samtools Pileup files using a separate, in-house validation script based on tumor and normal allele depth and quality. All confirmed variants were subjected to an additional, manual review.

### Identification of copy number variants

Copy number variation was determined primarily through ASCAT [35]. Only segments under 10 Mb in length were considered. Genes falling in these segment regions were annotated using the Ensembl variant effect predictor (Ensembl 64). Segment data were analyzed using R, Nexus Copy Number Software 6.1 (Biodiscovery) [56], visualized using IGV [57,58] and plotted using Progenetix [59].

#### Array comparative genomic hybridization

aCGH was performed on a subset of 24 zebrafish melanoma normal and tumor samples using a Nimblegen Custom Design 12 × 135 K CGH Array (Roche Nimblegen Technologies, catalogue number 05223881001 (Basel, Switzerland)) containing 135,000 probes covering the length of the zebrafish Zv9 genome. In brief, tumor and normal DNA were labeled, competitively hybridized to the array for 48 hours, washed and scanned using a 5 micron scanner (Molecular Devices (Sunnyvale, California, USA)). Signal intensities were extracted using the DEVA v1.2.1 Software (Nimblegen) [60]. Overall data quality was evaluated as recommended in the *DEVA Software User’s Guide*[61]. Segmentation was performed using the R Copynumber package [62] and visualized using the Nexus Copy Number Software (6.1) (Biodiscovery), IGV [57,58] and Progenetix [59].

### Statistical analyses

#### Codon selection

We used the method described in [63] to evaluate whether amino acid changes in ZD0038a occurred at a higher frequency than expected in the absence of positive selection. Briefly, we used 12 parameters to describe the different rates of the 12 possible single nucleotide substitutions, and two parameters (analogous to dN/dS) to describe selection at missense and nonsense mutations. This allowed us to quantify the strength of the selection without the confounding effect of sequence composition and different rates of each substitution type. Maximum-likelihood was used to estimate these parameters and likelihood ratio tests were used to test deviations from neutrality (dN/dS = 1). Analogous results to those presented in the main text were obtained using the traditional codon model approach used in phylogenetic analyses (implemented in [64]) as well as accounting for CpG context-dependent effects.

#### Estimation of the number of mutated copies

Allele-specific copy number estimates for point mutations were obtained by integrating copy number and sequencing data as described in [35].

#### Simulations of amplifications and homozygous deletions

Genes showing enrichment of amplifications were identified by permutation analysis, where 1,000,000 permutations were performed randomizing the positions (but not the size) of amplifications, for each sample. For each permutation and each gene, the number of samples that were hit by an amplification was counted and the probability that each gene was significantly enriched for amplifications was calculated as the proportion of the permutations in which that gene had as many, or more, amplifications than were observed in the ‘real’ data. Probabilities were adjusted for multiple testing using the Bonferroni correction (n = 6,677, the number of genes tested).

#### Evaluation of driver and age correlation

Mutation burden and driver correlation was performed as previously described [20].

### Pathway analysis

#### Mutation significance analysis

We combined mutation data from substitutions, insertions and deletions, and copy number changes (amplifications and homozygous deletions) to assess the likelihood of a gene being mutated in more samples than expected by chance. As each mutation type can occur at a different frequency (where amplifications are more frequent than deletions), each mutation type was considered separately. Thus, to calculate a combined *P*-value for each gene *j*, we used the following Equation 1:

(1) $$\begin{array}{l} p_{j}=\prod_{i\in \left\{1,2,3,4\right\}}P\left(X\geq x_{\mathrm{ij}}\left|q_{i},n_{i}\right)\right)\\ \;=\prod_{i\in \left\{1,2,3,4\right\}}\left(1-\sum_{k=0}^{k=x_{\mathrm{ij}}-1}\left({}_{k}^{n_{i}}\right){\left(q_{i}\right)}^{k}{\left(1-q_{i}\right)}^{n_{i}-k}\right), \end{array}$$

where *x*_*ij*_ is the number of samples carrying a mutation in gene *j* in sample group *i* and *n*_*i*_ the number of samples in sample group *i*. Moreover, *q*_*i*_ was calculated as follows:

$$q_{i}-\frac{1}{n_{i}}{\sum_{k=1}^{n_{i}}1-\left(1-\frac{1}{N}\right)}^{m_{k}},$$

with *N* number of genes in the genome.

#### Entrez gene mapping

For compatibility with the KEGG database, we mapped *Danio rerio* Ensembl IDs onto Entrez IDs using NCBI [65], which includes a cross-reference of Entrez to Ensembl. Target genes that could not be matched in this fashion were matched using gene symbol and synonyms.

#### Pathway analysis

We used knowledge from the KEGG database to construct a large protein interaction network. To gauge whether a pathway contains more frequently mutated genes than expected by chance, a KEGG pathway enrichment was performed for all 215 pathways in the *Danio rerio* specific KEGG database.

All genes with a combined *P*-value <0.05 (as calculated according to the mutation significance analysis) were selected for the pathway analysis. This cutoff selected for genes with at least two amplifications, and given rarer mutation types, genes with at least one mutation other than an amplification.

We called a gene frequently mutated if it carried at least *N* mutations, where *N* can be between 1 and 10 (Additional file 1: Table S9). Genes with mutation counts of three or more were visualized in the context of their KEGG pathway interactions using Cytoscape [66].

## Abbreviations

aCGH: Array comparative genomic hybridization; bp: Base pair; BWA: Burrows-Wheeler algorithm; GLM: Generalized linear model; KEGG: Kyoto Encyclopedia of Genes and Genomes; MAPK: Mitogen-activated protein kinase; PKA: Protein kinase A; SGA: String Graph Assembler; UTR: untranslated region.

## Competing interests

The authors declare that they have no competing interests.

## Authors’ contributions

JY designed the study, performed research, analyzed data and wrote the paper. RW, EEP, DS and PAF participated in the study design. DCW, PVL, JDR, IM, IW and CJW analyzed data and performed research. RW, AC, JR, CKK, EL, AMT, LC, LZ, JL, DJA and EEP contributed novel reagents. MD, JC and YM contributed graphic support. DRJ, JM, PT, KR, AB, JT and SM contributed to the data processing. SZ, MR, AS and PT contributed to the data analysis. LM, IW, SG, CL and SO contributed to the labwork. RW, DCW, PVL, JDR, IM, CK, DJA, LIZ, EEP, DS and PAF critically read the manuscript, which was approved by all the authors.

## Supplementary Material

Additional file 1: Tables S1 to S10 **Table S1:** zebrafish tumors used in the exome study. **Table S2:** sequencing coverage and metrics. **Table S3:** somatic mutations identified in the 53 zebrafish melanomas. **Table S4:** copy number changes identified in the 53 zebrafish melanomas. **Table S5: ***P*-values of genes occurring in amplifications. **Table S6: ***P*-values of genes occurring in homozygous deletions. **Table S7:** insertion and deletions identified in the 53 zebrafish melanomas. **Table S8:** statistical analysis of mutation burden correlation. **Table S9:** significance of genes with respect to frequency and modality. **Table S10** Mutated pathways and their significance from enrichment analysis. Click here for file

Additional file 2: Figures S1 to S8 **Figure S1:** effect of SNP density on germline and somatic substitution calling performance using CaVEMan. **Figure S2:** comparison of substitution calling algorithms on zebrafish melanoma data. **Figure S3:** experimental outline. **Figure S4:** evidence of two additional cluster of mutations in ZD8a on chromosome 10. **Figure S5:** comparison of copy number aberration profiles between ASCAT and aCGH. **Figure S6:** unsupervised clustering analysis of copy number aberrations. **Figure S7:** pathway analysis of all mutations. **Figure S8:** distribution of co-occurring copy number alterations and/or somatic mutations in *TP53*, *MITF*, and *CDKN2A* across 120 *BRAF* mutant melanomas identified in the SKCM TCGA dataset. Click here for file

Additional file 3 Supplementary text describing the mutation calling simulations and comparison of mutation callers. Click here for file
