# Supplementary material for: The genetic heterogeneity and mutational burden of engineered melanomas in zebrafish models
Source: Genome Biol. 2013 Oct 23;14(10):R113. doi: 10.1186/gb-2013-14-10-r113 (PMC3983654; doi:10.1186/gb-2013-14-10-r113)
Supplement: Additional file 2: Figures S1 to S8 — Figure S1: effect of SNP density on germline and somatic substitution calling performance using CaVEMan. Figure S2: comparison of substitution calling algorithms on zebrafish melanoma data. Figure S3: experimental outline. Figure S4: evidence of two additional cluster of mutations in ZD8a on chromosome 10. Figure S5: comparison of copy number aberration profiles between ASCAT and aCGH. Figure S6: unsupervised clustering analysis of copy number aberrations. Figure S7: pathway analysis of all mutations. Figure S8: distribution of co-occurring copy number alterations and/or somatic mutations in TP53, MITF, and CDKN2A across 120 BRAF mutant melanomas identified in the SKCM TCGA dataset. [file gb-2013-14-10-r113-S2.pdf]

Figure S1

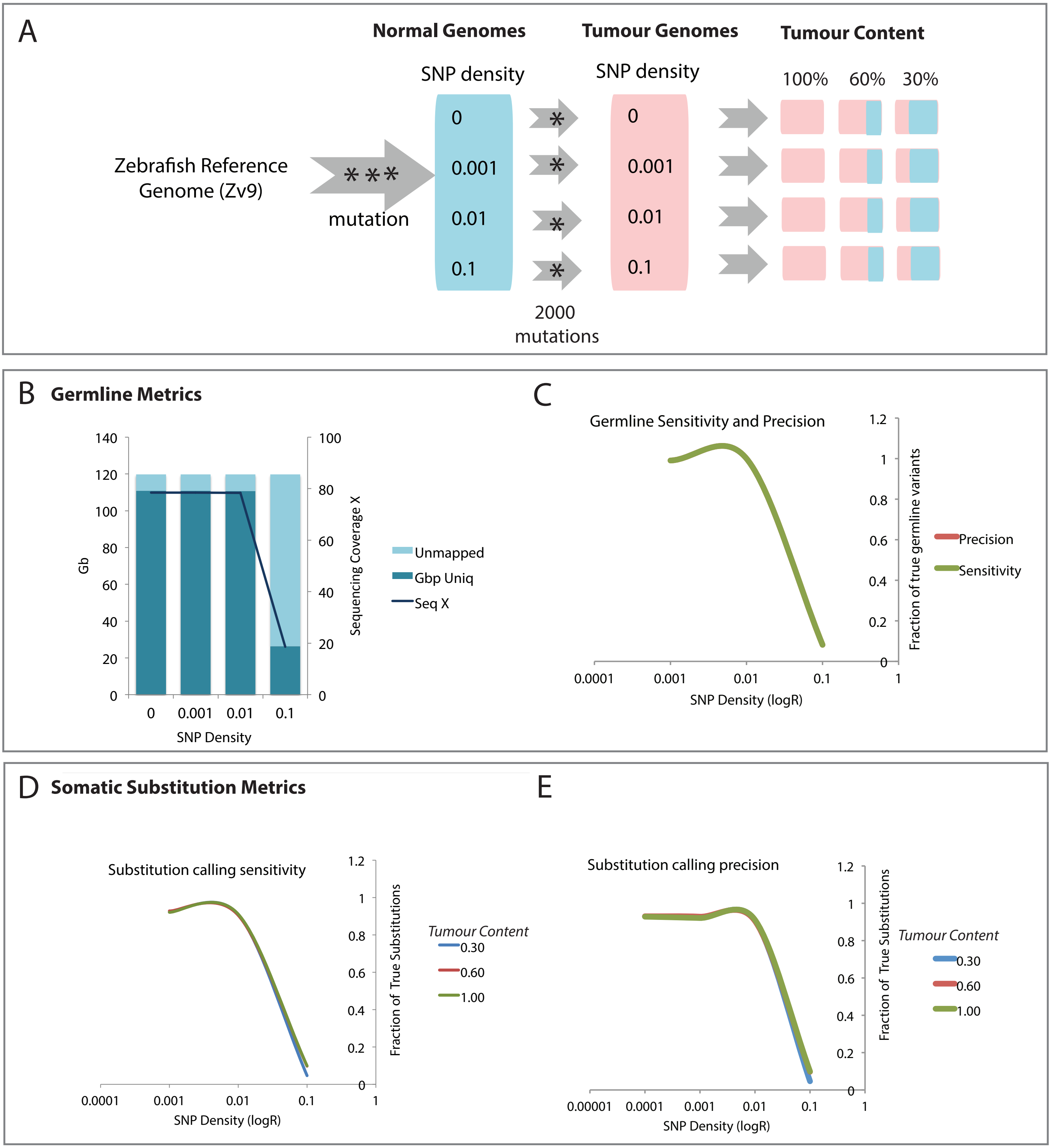
A
Normal Genomes
Tumour Genomes
Tumour Content
SNP density
SNP density
100%
60%
30%
Zebrafish Reference Genome (Zv9)
* * *
mutation
0
0.001
0.01
0.1
*
*
*
*
0
0.001
0.01
0.1
2000 mutations
B Germline Metrics
Gb
140
120
100
80
60
40
20
0
100
80
60
40
20
0
Sequencing Coverage X
0
0.001
0.01
0.1
SNP Density
Unmapped
Gbp Uniq
Seq X
C
Germline Sensitivity and Precision
1.2
1
0.8
0.6
0.4
0.2
0
Fraction of true germline variants
0.0001
0.001
0.01
0.1
1
SNP Density (logR)
Precision
Sensitivity
D Somatic Substitution Metrics
Substitution calling sensitivity
1.2
1
0.8
0.6
0.4
0.2
0
Fraction of True Substitutions
0.0001
0.001
0.01
0.1
1
SNP Density (logR)
Tumour Content
0.30
0.60
1.00
E
Substitution calling precision
1.2
1
0.8
0.6
0.4
0.2
0
Fraction of True Substitutions
0.00001
0.0001
0.001
0.01
0.1
1
SNP Density (logR)
Tumour Content
0.30
0.60
1.00

Figure S2

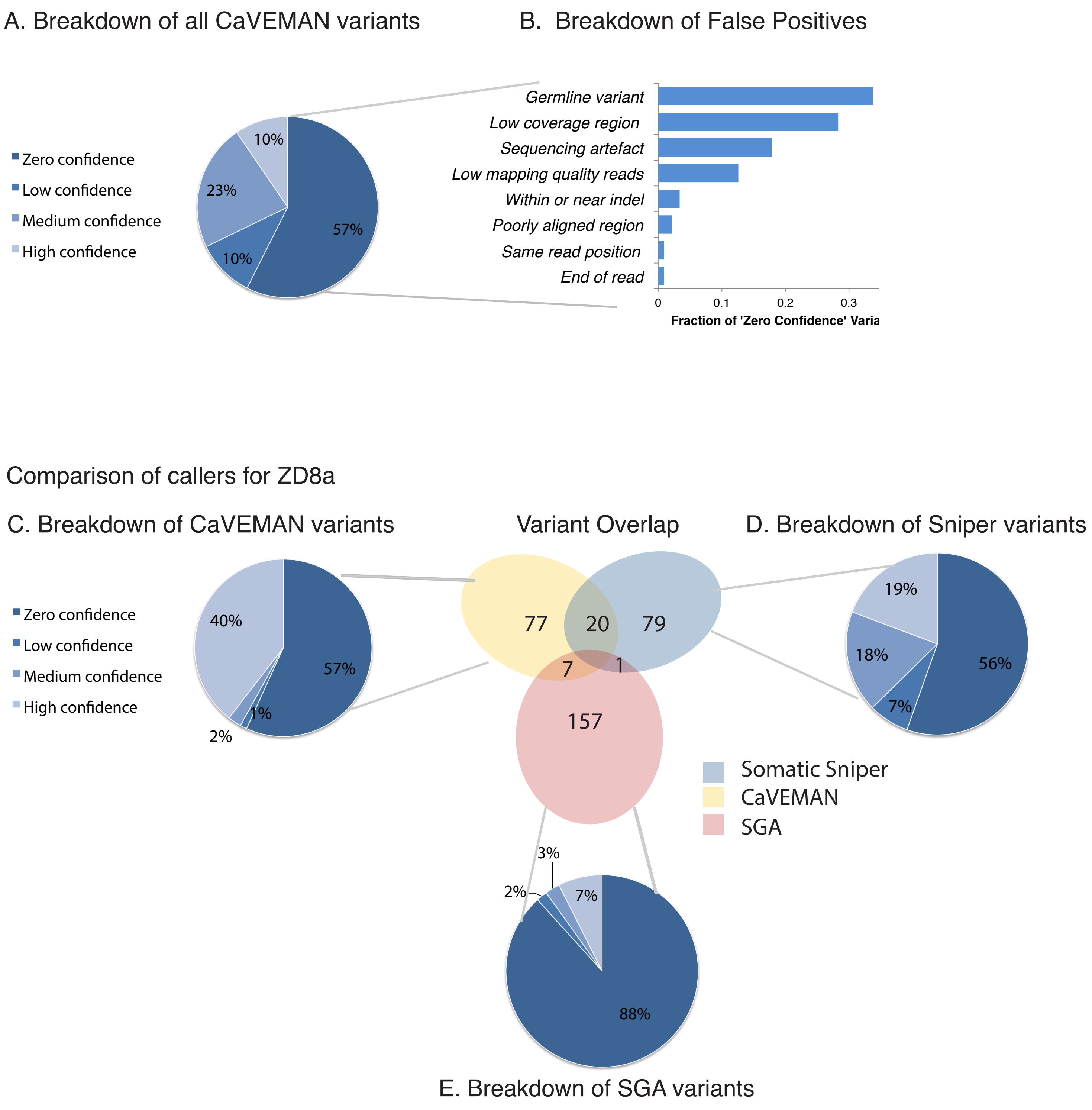
A. Breakdown of all CaVEMAN variants
Zero confidence
Low confidence
Medium confidence
High confidence
10%
23%
57%
10%
B. Breakdown of False Positives
Germline variant
Low coverage region
Sequencing artefact
Low mapping quality reads
Within or near indel
Poorly aligned region
Same read position
End of read
0
0.1
0.2
0.3
Fraction of 'Zero Confidence' Varia
Comparison of callers for ZD8a
C. Breakdown of CaVEMAN variants
Zero confidence
Low confidence
Medium confidence
High confidence
40%
57%
1%
2%
Variant Overlap
77
20
79
7
1
157
Somatic Sniper
CaVEMAN
SGA
D. Breakdown of Sniper variants
19%
18%
56%
7%
3%
2%
7%
88%
E. Breakdown of SGA variants

Figure S3

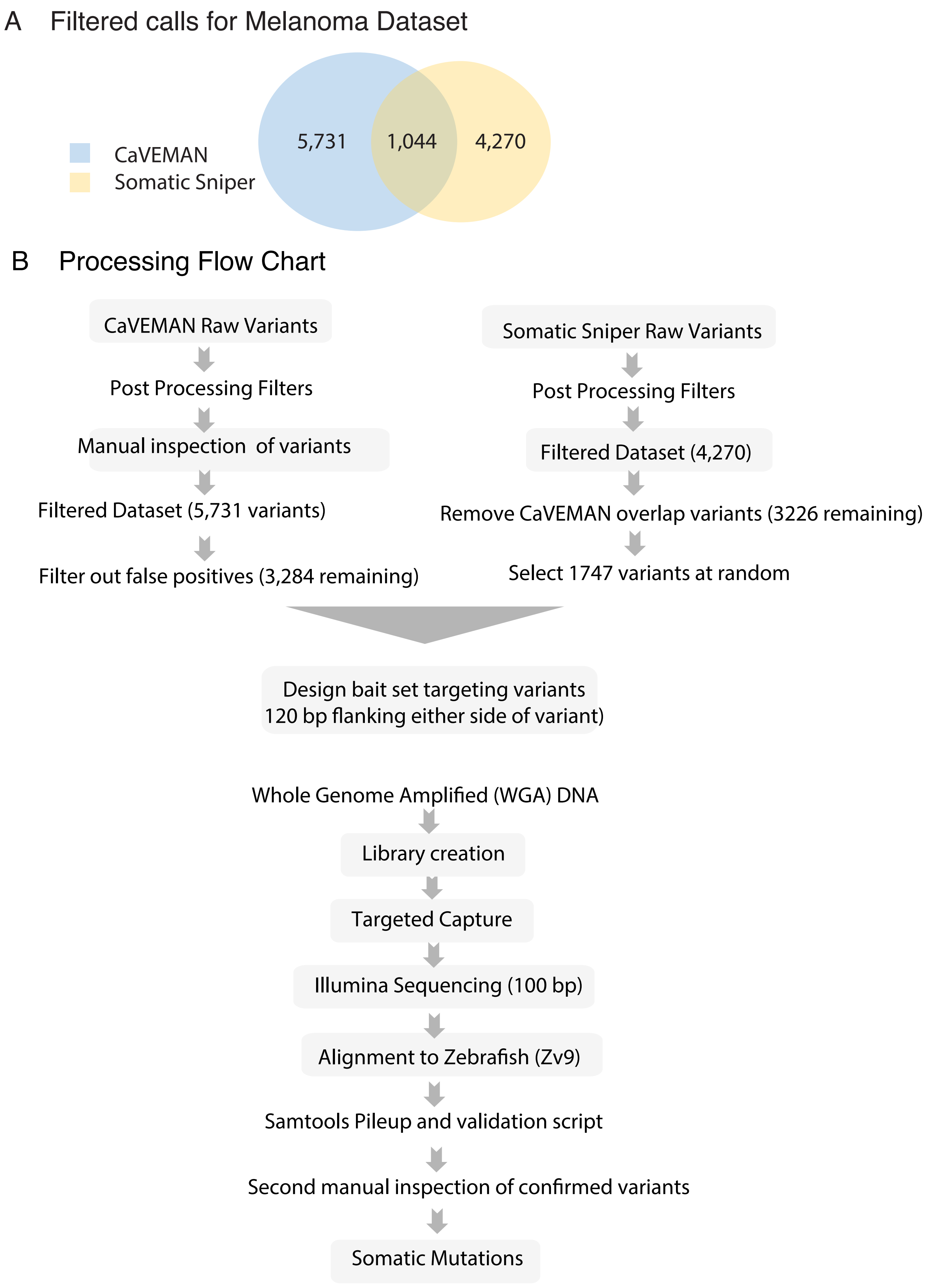
A Filtered calls for Melanoma Dataset
CaVEMAN
Somatic Sniper
5,731
1,044
4,270
B Processing Flow Chart
CaVEMAN Raw Variants
Post Processing Filters
Manual inspection of variants
Filtered Dataset (5,731 variants)
Filter out false positives (3,284 remaining)
Somatic Sniper Raw Variants
Post Processing Filters
Filtered Dataset (4,270)
Remove CaVEMAN overlap variants (3226 remaining)
Select 1747 variants at random
Design bait set targeting variants
120 bp flanking either side of variant)
Whole Genome Amplified (WGA) DNA
Library creation
Targeted Capture
Illumina Sequencing (100 bp)
Alignment to Zebrafish (Zv9)
Samtools Pileup and validation script
Second manual inspection of confirmed variants
Somatic Mutations

Figure S4

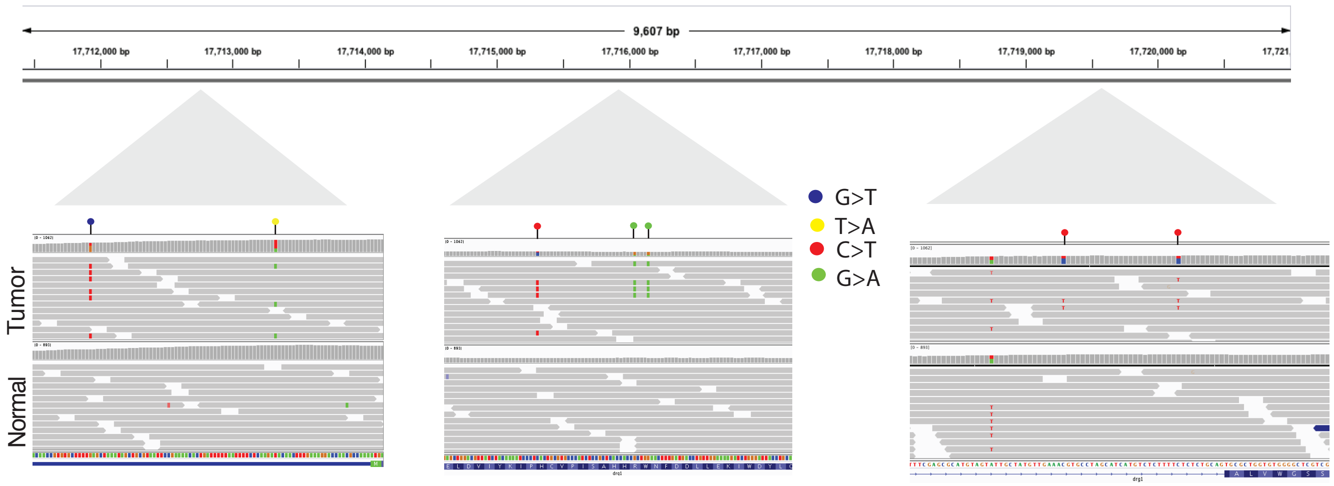
9,607 bp
17,712,000 bp
17,713,000 bp
17,714,000 bp
17,715,000 bp
17,716,000 bp
17,717,000 bp
17,718,000 bp
17,719,000 bp
17,720,000 bp
G>T
T>A
C>T
G>A
Tumor
Normal

Figure S5

A *NRAS;p53-/-;X*

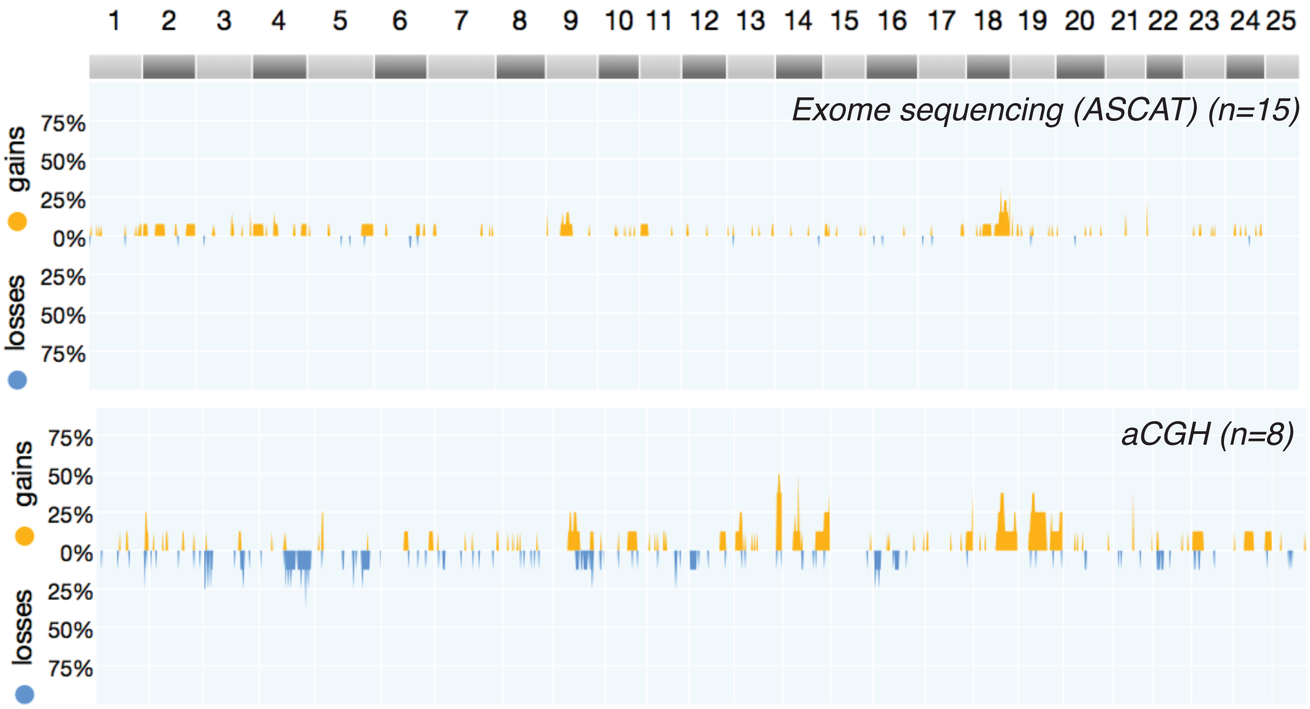

B *BRAF^{V600E};mitf-/-;p53-/-;mitf:MITF;mitf:X*

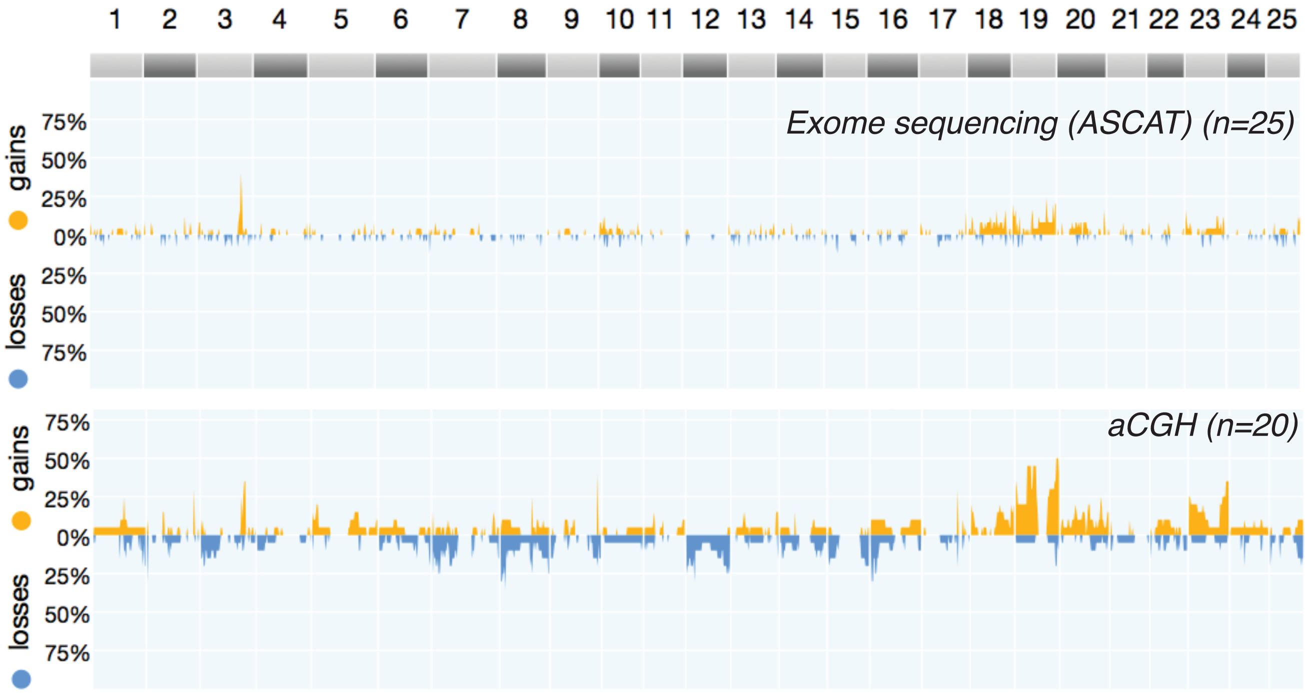

Figure S6

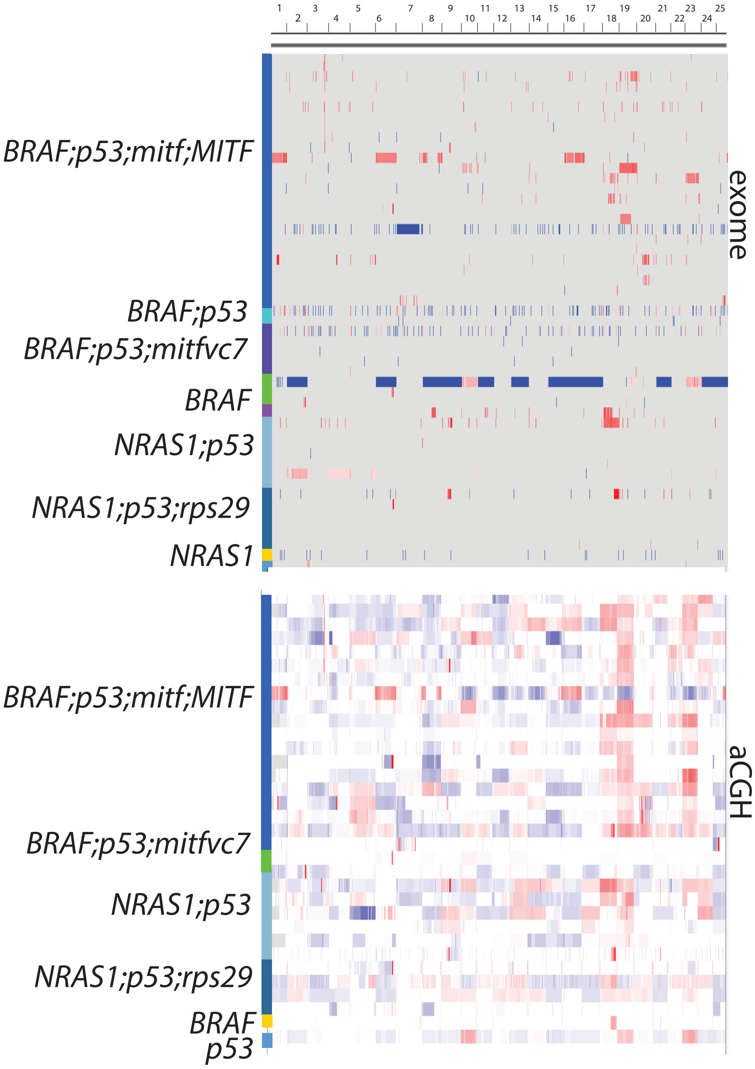

1 2 3 4 5 6 7 8 9 10 11 12 13 14 15 16 17 18 19 20 21 22 23 24 25
BRAF;p53;mitf;MITF
BRAF;p53
BRAF;p53;mitfvc7
BRAF
NRAS1;p53
NRAS1;p53;rps29
NRAS1
exome
BRAF;p53;mitf;MITF
BRAF;p53;mitfvc7
NRAS1;p53
NRAS1;p53;rps29
BRAF
p53
aCGH

Figure S7

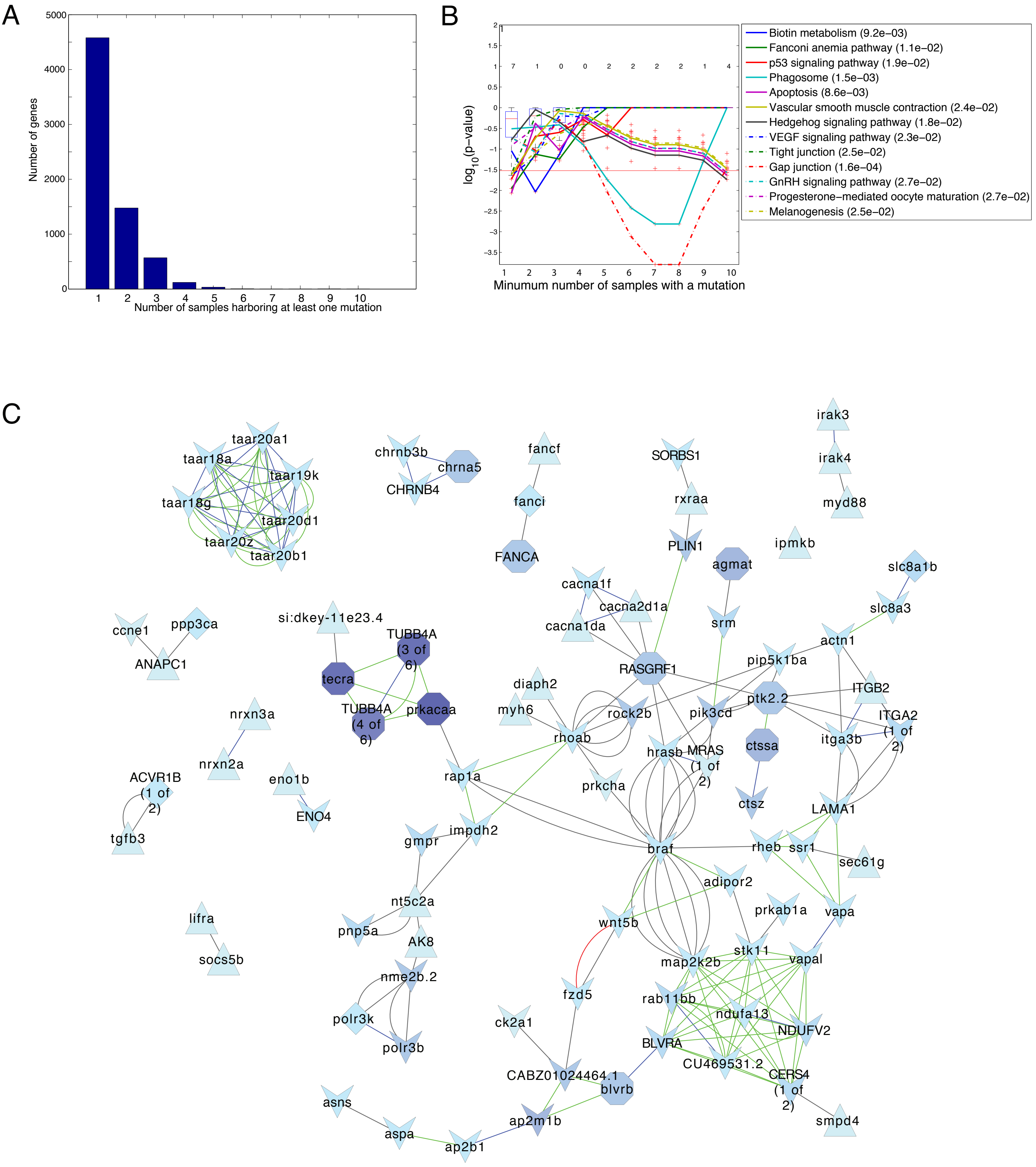
A
Number of genes
Number of samples harboring at least one mutation
5000
4000
3000
2000
1000
0
1
2
3
4
5
6
7
8
9
10
B
log10(p−value)
Minumum number of samples with a mutation
7
1
0
0
2
2
2
2
1
4
Biotin metabolism (9.2e−03)
Fanconi anemia pathway (1.1e−02)
p53 signaling pathway (1.9e−02)
Phagosome (1.5e−03)
Apoptosis (8.6e−03)
Vascular smooth muscle contraction (2.4e−02)
Hedgehog signaling pathway (1.8e−02)
VEGF signaling pathway (2.3e−02)
Tight junction (2.5e−02)
Gap junction (1.6e−04)
GnRH signaling pathway (2.7e−02)
Progesterone−mediated oocyte maturation (2.7e−02)
Melanogenesis (2.5e−02)
C
taar20a1
taar18a
taar19k
taar18g
taar20d1
taar20z
taar20b1
chrnb3b
chrna5
CHRNB4
fancf
fanci
FANCA
SORBS1
rxraa
PLIN1
irak3
irak4
myd88
ipmkb
agmat
slc8a1b
slc8a3
cacna1f
cacna2d1a
cacna1da
srm
ccne1
ppp3ca
ANAPC1
si:dkey-11e23.4
TUBB4A (3 of 6)
tecra
TUBB4A (4 of 6)
prkacaa
RASGRF1
actn1
pip5k1ba
ptk2.2
ITGB2
ITGA2 (1 of 2)
diaph2
myh6
rock2b
pik3cd
rhoab
ctssa
itga3b
nrxn3a
nrxn2a
hrasb
MRAS (1 of 2)
eno1b
ENO4
ACVR1B (1 of 2)
tgfb3
rap1a
prkcha
ctsz
LAMA1
gmpr
impdh2
braf
rheb
ssr1
sec61g
adipor2
nt5c2a
prkab1a
vapa
lifra
socs5b
pnp5a
wnt5b
AK8
map2k2b
stk11
vapal
nme2b.2
fzd5
rab11bb
ndufa13
polr3k
polr3b
ck2a1
BLVRA
NDUFV2
CU469531.2
CERS4 (1 of 2)
CABZ01024464.1
blvrb
smpd4
asns
ap2m1b
aspa
ap2b1

Figure S8

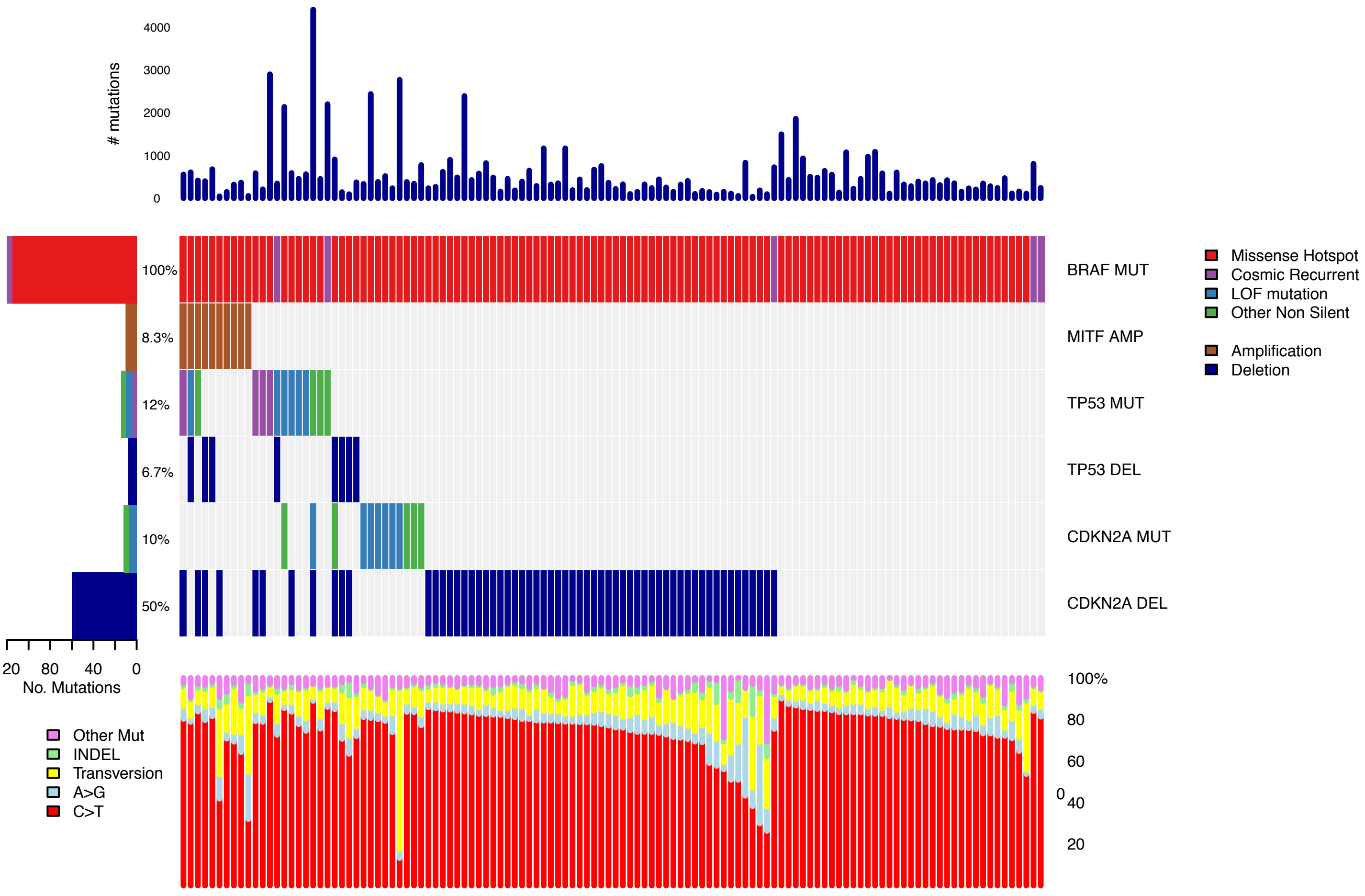

mutations
4000
3000
2000
1000
0
100%
8.3%
12%
6.7%
10%
50%
BRAF MUT
MITF AMP
TP53 MUT
TP53 DEL
CDKN2A MUT
CDKN2A DEL
Missense Hotspot
Cosmic Recurrent
LOF mutation
Other Non Silent
Amplification
Deletion
120 80 40 0
No. Mutations
Other Mut
INDEL
Transversion
A>G
C>T
100%
80
60
0
40
20
