# Supplementary material for: The genetic heterogeneity and mutational burden of engineered melanomas in zebrafish models
Source: Genome Biol. 2013 Oct 23;14(10):R113. doi: 10.1186/gb-2013-14-10-r113 (PMC3983654; doi:10.1186/gb-2013-14-10-r113)
Supplement: Additional file 3 — Supplementary text describing the mutation calling simulations and comparison of mutation callers. [file gb-2013-14-10-r113-S3.docx]

**SUPPLEMENTARY TEXT**

**Assessment of the amenability of zebrafish genome to mutation calling algorithms**

To determine the amenability of zebrafish genomic features to standard sequence alignment and downstream mutation calling, we started our analysis by measuring the performance of BWA alignment and mutation calling using CaVEMAN (Cancer Variant detection through Expectation Maximization), an in-house substitution calling algorithm for human cancers. Four individual zebrafish genomes that differed in levels of SNP densities ranging from 0, 0.001 (comparable to human), 0.01 (comparable to zebrafish), and 0.1 SNPs/kb were constructed from the reference genome using an in-house mutation simulator (Figure S1a). An additional 2,000 mutations were introduced to each individual to create a matching “tumour”, resulting in a total of 4 tumour-normal genome pairs. Seventy-five base pair (bp) paired-end reads were generated and aligned back to the reference to achieve an average coverage of 80X per genome.

Measurements of alignment quality based on sequencing coverage, proportion of uniquely mapping and high quality mapping reads (mapping quality over 40) were high and unaffected by diversity up to 0.1 SNPs/base, thus revealing a high fidelity of the zebrafish genome alignment back to the reference. At 0.1 SNPs/base, a three-fold reduction in these metrics was observed (Figure S1b). We tested the sensitivity and precision of CaVEMAN for identifying both germline and somatic alleles in these simulated tumour-normal pairs. As with mapping metrics, precision and sensitivity of identifying germline variants in these simulations were optimal (nearly 100%) for SNP densities approaching 0.1 SNPs/base, after which sensitivity and precision abruptly declined (Figure S1c).

For somatic variants, we applied CaVEMAN and standard post-processing filters used for human genomes to the zebrafish dataset to obtain a high confidence set of somatic substitutions. Given the frequent contamination of tumour samples with normal tissue, we had constructed tumour genomes to reflect up to 70% of normal contamination. Sensitivity of substitution calling by CaVEMAN was high (nearly 90%) for samples with SNP densities up to 0.01 but declined to less than 10% at 0.1 SNPs/base (Figure S1d). Normal contamination did not appear to affect the sensitivity or precision of substitution calling in combination with SNP densities up to this threshold (Figure S1d). Together, the data demonstrated that alignment, germline and substitution calling are unaffected to levels of diversity presenting in zebrafish genomes with up to 70% normal contamination under controlled simulation conditions. It is worth noting however that only homozygous mutant alleles were considered in these simulations, and other types of germline variation (including repeats, rearrangements and insertions and deletions) were not evaluated and may also contribute significantly to the quality of sequence alignment and mutation calling.

Comparison of CaVEMAN with Somatic Sniper and String Graph Assembler

Processing with Somatic Sniper (Sniper) and in-house filters resulted in a comparable number of calls for this sample (79 vs 77, Figure S2d). Manual inspection revealed a greater proportion of high confidence variants for overlapping variants than variants for either caller alone (71% vs 19% respectively. Figure S2e). Whilst SNIPER missed 21 high confidence CaVEMAN variants from the filtered set, it called 9 new medium to high confidence variants, indicating that inclusion of Sniper could heighten the sensitivity of our analysis. We next employed SGA, which was compared to Somatic Sniper and a prototype of an assembly-based variant caller based on the SGA assembler. SGA directly compares sequence reads between the tumour and normal pair, which would in theory reduce the number of artifacts resulting from the misalignment of short reads spanning polymorphisms in the reference genome. In this algorithm, short substrings of a uniform length unique to the tumour are assembled to describe candidate variants with respect to the normal. Haplotypes in this contig are derived and aligned back to the reference.

The prototype SGA caller made 162 substitution calls, 88% of which were determined to be germline variants by manual inspection (Figure S2e. Of the remaining 19 non-germline variants, 12 were determined to be high-confidence substitutions. The assembly caller missed 28 high-confidence substitutions found by Somatic Sniper or CaVEMAN, and was not considered in the subsequent analysis.
